# Epidemiology of Carbapenem Resistant *Klebsiella pneumoniae* Infections in Mediterranean Countries

**DOI:** 10.4084/MJHID.2016.032

**Published:** 2016-07-01

**Authors:** Corrado Girmenia, Alessandra Serrao, Martina Canichella

**Affiliations:** Dipartimento di Ematologia, Oncologia, Anatomia Patologica e Medicina Rigenerativa, Azienda Policlinico Umberto I, Sapienza University of Rome, Rome, Italy

## Abstract

Infections by Carbapenem-Resistant Enterobacteriaceae (CRE), in particular, carbapenem-resistant *Klebsiella pneumoniae* (CRKp), are a significant public health challenge worldwide. Resistance to carbapenems in enterobacteriaceae is linked to different mechanisms, including the production of the various types of enzymes like KPC, VIM, IMP, NDM, and OXA-48. Despite several attempts to control the spread of these infections at the local and national level, the epidemiological situation for CRKp had worsened in the last years in the Mediterranean area. The rate and types of CRKp isolates greatly differ in the various Mediterranean countries. KPC-producing *K. pneumoniae* is diffused particularly in the European countries bordering the Mediterranean Sea and is endemic in Greece and Italy. On the contrary, OXA-48-producing *K. pneumoniae* is endemic in Turkey and Malta and diffused at inter-regional level particularly in some

North African and Middle East countries. The spread of these multiresistant pathogens in the world and the Mediterranean countries has been related to various epidemiological factors including the international transfer of patients coming from endemic areas.

## Introduction

Infections by Carbapenem-Resistant Enterobacteriaceae (CRE), in particular carbapenem-resistant *Klebsiella pneumoniae* (CRKp), are a significant public health challenge worldwide.^1^–^4^ These pathogens are characterized by multiantibiotic resistance which involves penicillins, all cephalosporins, monobactams, carbapenems, and even β-lactamase inhibitors. They are generally only susceptible to a few antibiotics, and there is high mortality among patients with bloodstream infections caused by these organisms. Many CRKp isolates remain susceptible to colistin, tigecycline, and one or more aminoglycoside, but some are resistant even to these drugs.^5^,^6^ Moreover, only a few drugs are in development against CRE.^7^ The prognosis of infections by CRKp is particularly poor in high risk immunocompromised populations as intensive care unit (ICU), solid organ transplant (SOT), hematological malignancies (HEM) and stem cell transplant (SCT) patients.^8^–^15^

Resistance to carbapenems in enterobacteriaceae is linked to different mechanisms, in particular the production of strong carbapenemases, but also of beta-lactamases that possess weak carbapenemase activity when combined with decreased permeability due to porin loss or alteration. Strong carbapenemases that are responsible for nonsusceptibility to carbapenems, without additional permeability defects, belong to Ambler molecular class A, B, or D. These enzymes are carried either on chromosome or acquired via plasmids.^16^,^17^

*K. pneumoniae* carbapenemase (KPC) enzymes are currently the most clinically significant enzymes among the class A carbapenemases. KPC producing *K. pneumoniae* had the widest dissemination in the last few years being diffused with important regional, interregional and endemic spread in several countries. To date, more than 20 different KPC variants have been described, even though KPC-2 and -3 remain the most commonly identified variants.^18^ The class B beta-lactamases or metallo-beta-lactamases (MBLs) have also been identified in various enterobacterial species, including *K. pneumoniae*.^19^ They are mainly New Delhi metallo-beta-lactamase (NDM-1), Verona integron-encoded metallo-beta-lactamase (VIM), and Imipenemase (IMP) type enzymes, with the first group being the most commonly identified worldwide. Carbapenem-hydrolysing oxacillinase-48 (OXA-48) is the most frequently reported class D beta-lactamase and microorganisms producing this enzyme have almost reached the same spread of KPC-producing *K. pneumoniae*.^17^,^19^,^20^

In 2012, the European Centre for Disease Prevention and Control (ECDC) launched the ‘European survey of carbapenemase-producing Enterobacteriaceae (EuSCAPE)’ project to gain insights into the occurrence and epidemiology of CRE and to increase the awareness of the spread of CRE, in Europe.^21^ The epidemiological situation for CRKp had worsened since 2010 and CRKp continued to spread in European hospitals, particularly in the Mediterranean area.

The aim of this review is to describe the recent epidemiological data on the diffusion of CRKp infections in Mediterranean countries.

## Epidemiology of CRKp in Mediterranean countries

**Figure 1:**
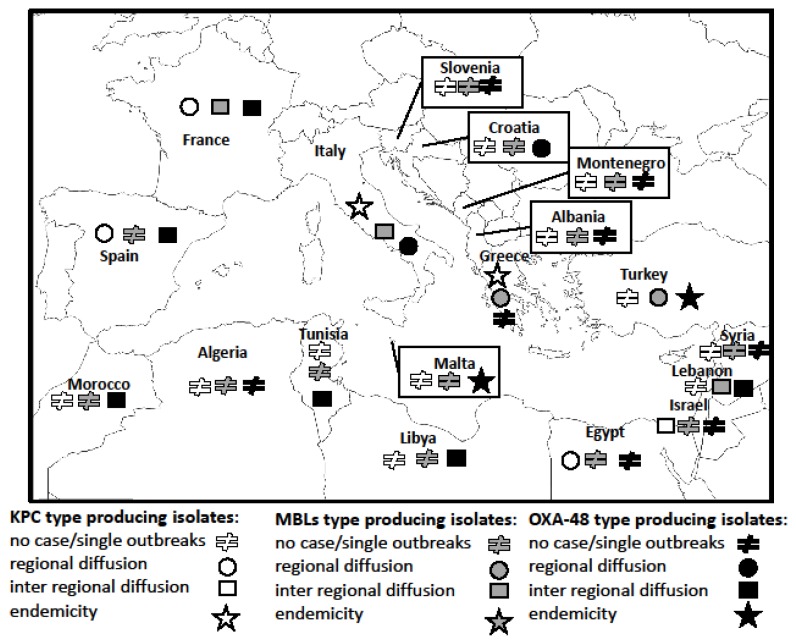
Current epidemiological stages of carbapenem-resistant *Klebsiella pneumoniae* by type of carbapenemase in the Mediterranean countries

The epidemiology of the various types of CRKp is characterized by a distribution which does not necessarily overlap in the different countries (Figure 1). It is well defined in Europe and Israel where several national and multinational epidemiological surveys have been performed in the last years, on the contrary, the data from North African and Middle East regions, other than Israel, are less accurate.^22^

### Class A KPC type producing K. pneumoniae

KPC was initially reported from *K. pneumoniae* strains isolated in the United States in the late 1990s. KPC producing *K. pneumoniae* has since spread across the United States and the overall prevalence of carbapenem resistance among *Klebsiella* spp. isolates causing hospital-acquired infections in U.S. hospitals was approximately 12% between 2009 and 2010.^23^–^29^ KPC-producing *K. pneumoniae* has since spread worldwide. The first country besides the United States that experienced a nationwide outbreak was Israel in late 2005 where the pathogen rapidly became endemic.^30^,^31^ It was the first outbreak of KPC-producing K. pneumoniae in the Mediterranean area, presumably coming from U.S. Hospital-level interventions were implemented but were unable to contain its spread, therefore in 2007 the Israeli Ministry of Health launched a nationwide intervention, which in few months was able to contain the outbreak nationally, with a 79% relative reduction in the incidence compared with its peak the previous year. In Israel KPC-positive *K. pneumoniae* was declared as an endemic phenomenon until 2010, while, thanks to the nationwide infection-control intervention, since 2011 the epidemiological scale downgraded to inter-regional spread.^32^

Since the Israeli epidemic, several sporadic cases and outbreaks of KPC type *K. pneumoniae* isolates have been reported in many European countries. In 2007 two cases of KPC-2 positive *K. pneumoniae* infection were diagnosed in France and Sweden, respectively, but both cases were documented in patients transferred from Greece.^33^,^34^ In the same year an outbreack of KPC-2-positive *K. pneumoniae* was reported from a tertiary hospital in Crete and within 2 years of these reports, the multiresistant pathogen disseminated into all Greek tertiary-care hospitals, not only in Intensive Care Units, but also in medical and surgical wards.^35^–^41^ According to the data of the European Antimicrobial Resistance Surveillance Network (EARS-Net) of the European Centre for Disease Prevention and Control (ECDC) the epidemiology of KPC-positive *K. pneumoniae* in Greece was considered at an endemic stage since 2010, and since 2011 the rate of resistance to carbapenems (mainly due to KPC production) was constantly over 60% in invasive *K. pneumoniae* isolates.^42^

In Italy, the first KPC-positive *K. pneumoniae* strains were isolated in 2008 from an inpatient with a complicated intra-abdominal infection in Florence and in 2009 from two patients admitted to a teaching hospital in Rome.^43^,^44^ Since then several cases and outbreaks by KPC-2 and KPC-3 *K. pneumoniae* strains were reported as a consequence of a rapid dissemination in almost all hospitals in the north, middle and south regions of the country.^8^,^9^,^13^,^14^,^45^–^51^ KPC-positive *K. pneumoniae* have spread rapidly and extensively in Italy, with a sharp increase reported by the EARS-Net surveillance system for bacteraemia isolates, from 1–2% carbapenem resistance in 2006–09 to 26.7% in 2011 and 32.9% in 2014. Since 2013, in Italy the epidemiology of KPC-positive *K. pneumoniae* was declared at an endemic stage.^42^

The first two KPC positive *K. pneumoniae* clinical isolates (one KPC-2 and one KPC-3) in France were found in 2005.^52^,^53^ They were recovered from two patients with a recent hospital admission in New York City (USA). A further case was described two years later from a patient transferred from a Greek hospital (see above).^33^ Subsequently, other nosocomial clusters have been reported, generally related to patients transferred from other European endemic areas.^54^,^55^

In 2012, a national reference centre was established to examine all carbapenem-resistant Enterobacteriaceae in France. Throughout 7 months, out of 160 carbapenemase-positive Enterobacteriaceae identified, 19 (11.9%) KPC-producing *K. pneumoniae* cases were documented.^1^ Five of these 19 cases were linked to recent stays in Israel, Italy, Kuwait, and China, none was community acquired. According to the EARS-Net data, in 2014 less than 1% of the invasive *K. pneumoniae* isolates in France were resistant to carbapenems and sporadic hospital outbreaks with no autochthonous inter-institutional transmission have been reported.^42^,^56^

The first eight cases of KPC-3-positive *K. pneumoniae* colonizations reported in Spain have been documented in 2009.^57^ These patients were in five different wards in a hospital in Madrid, and none had recently travelled to KPC-endemic countries. Since then, sporadic KPC-positive isolates have been identified in the same hospital and in other hospitals in Valencia. A slight increase of KPC-positive *K. pneumoniae* invasive isolates was observed in the following years in Spain as documented by the EARS-Net surveillance (from 0.3% in 2011 to 2,3% in 2014). In a multicentre prospective epidemiological study carried out in 83 Spanish hospitals from February to May 2013, out of 374 *K. pneumoniae* isolates 282 (74.4%) were carbapenemase producers but in only 3 (0.1%) cases the carbapenem resistance was related to KPC production.^58^,^59^ In the period 2014–2015, KPC-positive *K. pneumoniae* was reported at regional stage almost exclusively in the territory of Madrid and Valencia.^56^

To date, only sporadic epidemiological data on KPC-positive *K. pneumoniae* isolates have been reported in other European countries bordering the Mediterraneum. Forty-eight KPC-2 producing *K. pneumoniae* isolates, collected during a period from February 2011 to August 2013, were recorded in 9 of 40 centers in Croatia, limited to the northwest region of the country.^60^,^61^ According to the EARS-Net data, in 2014 0.9% of the invasive *K. pneumoniae* isolates in Croatia and Slovenia were resistant to carbapenems.^42^

A KPC-3 positive *K. pneumoniae* strain was isolated in Albania in 2014 in an ICU patient with no history of recent travel to endemic areas.^62^ According to the EuSCAPE data no case of KPC-positive *K. pneumoniae* has been reported in 2014–2015 from Montenegro, Malta and Turkey.

With regard to North African and the Middle East regions (excluding Israel), in most of the countries, there is no description or only single cases of KPC producers *K. pneumoniae* isolates.^22^ A case of KPC-3-producing *K. pneumoniae* meningitis in a 6-month-old child has been reported in Algeria.^63^ Three studies from tertiary care hospitals in Egypt showed a high prevalence of KPC related carbapenem non-susceptible *K. pneumoniae* (70% in one study) isolates suggesting an underestimated epidemiological phenomenon in that country.^64^–^66^

### Class B MBLs type producing K. pneumoniae

Class B MBLs are mostly of the VIM and IMP types and, more recently, of NDM-1 type.^67^ IMP-type beta-lactamases were the first acquired MBLs to be identified. They have been detected in a series of clinically relevant Gram-negative bacilli including *K. pneumoniae* but with a much lower frequency compared all other carbapenemases. IMP producing *K. pneumoniae*, which is dominant in the Asian continent and Australia, has been only sporadically detected in Europe and the Mediterranean area.^22^

The VIM type MBL is the most commonly found class B carbapenemase which has been identified in all continents.^19^,^68^ Italy was the first Mediterranean country to report acquired MBLs, with sporadic isolates of VIM-4-producing *K. pneumoniae*.^69^ Since then, single or sporadic hospital outbreaks caused by VIM-1 like enzymes were described from various regions in this country.^70^,^71^ VIM producing *K. pneumoniae* have not undergone wide dissemination in the other Mediterranean countries, with the exception of Greece where several cases and outbreaks have been reported in the last years.^67^,^72^ Finally, single reports and local outbreaks of VIM- type producers have been reported in other countries of the Mediterranean area, such as France,^73^ Spain,^58^ Morocco,^74^ Egypt,^75^ Algeria,^76^ and Tunisia.^77^

The NDM-1 enzyme is a more recently reported class B carbapenemase identified mostly in *E.coli* and *K. pneumoniae* and to a lesser extent in other enterobacteriaceae. NDM-1 producers have been reported in the environment and in the community. It arose from India in 2008 and spread rapidly over Indian subcontinent in the following few years and international travel has a significant impact on its rapid worldwide spread.^19^,^78^,^79^ The European epidemiology for CRE changed between 2013 and 2015 for NDM-producing Enterobacteriaceae with several countries reporting regional or inter-regional spread but with no endemic situation. Single or sporadic hospital outbreaks caused by NDM-1 producing *K. pneumoniae* strains were reported from many countries in the Mediterranean area: France,^80^ Italy,^81^ Spain,^82^,^83^ Morocco^84^ Tunisia in a patient transferred from Libya,^85^ and Egypt.^86^ In these countries the dissemination of NDM-producing CRE, including *K. pneumoniae*, was still limited and cases were mostly acquired abroad. Finally, an emergence of autochthonous and imported NDM-1 producing *K. pneumoniae* was reported in Turkey, especially in hospitals from cities close to the Syrian border, and in Greece.^87^–^90^ Sporadic cases or single hospital outbreaks by NDM-1 positive *K. pneumoniae* have been observed in Lebanon (imported from Iraq), Israel and other Middle East Mediterranean countries.^91^–^93^

### Class D OXA-48 type producing K. pneumoniae

Class D beta-lactamases, also named OXAs for oxacillinases, have more than 440 known variants with 232 of them showing carbapenemase activity. OXA-48 represents the main enzyme isolated around the world.^17^ It was initially identified in *K. pneumoniae* in a strain from Istambul, Turkey in 2001.^94^ The OXA-48-positive *K. pneumoniae* strains rapidly diffused in Turkey with hospital outbreaks in the main cities of the country.^95^ The epidemic was under controlled and in 2015 OXA-48 *K. pneumoniae* was considered endemic in Turkey.^56^

Identification of this novel and powerful resistance determinant outside of Turkey indicated that spread was larger than expected. OXA-48 producing isolates disseminated to the Middle East, North Africa and Europe and several nosocomial outbreaks were reported from many Mediterranean countries including France, Spain, Lebanon, Israel, Tunisia and Morocco.^97^–^108^ Several cases of OXA-48 *K. pneumoniae* infections in Libyan refugees transferred to hospitals of European countries have been reported, suggesting that the epidemiology of such multiresistant microorganisms in Libya, and probably in other north African countries, is underestimated.^109^–^113^ Dramatic epidemiological findings occurred in Malta where dissemination of OXA-48-producing Enterobacteriaceae had changed the country’s epidemiological level from rare sporadic occurrence before 2010 to an endemic situation by 2013.^42^,^56^ Presumably, the influx of injured Libyan war victims to the country’s only tertiary care hospital in 2011 contributed to the first outbreak and spread of OXA-48-producing Enterobacteriaceae in the country. Despite initial control of the outbreak, the situation rapidly became endemic in this hospital and OXA-48-producing *K. pneumoniae* and other enterobacteria spread to other health and residential care entities on the Maltese islands. EARS-Net data for Malta showed an increase in the percentage of invasive OXA-48–producing *K. pneumoniae* in Malta, from 3.8% in 2011, to 5.8% in 2013 and 9.9% in 2014.^42^

In summary, in 2014–2015 the epidemiological stage for the spread of OXA-48 producing *K. pneumoniae* in the Mediterranean area was: endemic level in Turkey and Malta, inter-regional diffusion in France, Spain, Lebanon, Tunisia, Morocco and Libya, regional diffusion in Italy and Croatia, single or sporadic hospital outbreaks in Israel, Slovenia, Albania, single cases or never reported in Greece, Albania, Montenegro, Syria, Egypt and Algeria.

## Conclusions

Despite several attempts to control the spread of CRKp at the local and national level, the epidemiological situation for these multiresistant pathogens had worsened in the last years in the Mediterranean area. The rate and types of CRKp isolates greatly differ in the various countries being the diffusion of these multiresistant pathogens related to various geographic and epidemiological factors including the international transfer of patients coming from endemic areas.

The political and economic relationship and the increasing phenomenon of migration in the Mediterranean basin should lead to a continuous epidemiological survey of multiresistant bacteria in order to define and plan appropriate infection-control intervention at national and international level.
